# A system for the management of sandy shorelines under climate change: United States Virgin Islands (USVI)

**DOI:** 10.1007/s13280-023-01946-w

**Published:** 2023-11-30

**Authors:** Theodoros Chalazas, Gerald Bove, Dimitrios Chatzistratis, Isavela N. Monioudi, Adonis F. Velegrakis

**Affiliations:** 1 Department of Marine Sciences, University of the Aegean, University Hill, 81100 Mytilene, Greece; 2 SUNY Oneonta Biological Field Station, 5838 State Hwy 80, Cooperstown, NY 13326 USA

**Keywords:** Beach erosion, Caribbean, Climate variability and change, Coastal Information and Assessment System, Integrated coastal zone management, GIS

## Abstract

A methodological framework is presented for the assessment of beach vulnerability to climate variability and change on small touristic islands. Based on the development of a coastal vulnerability index (CVI) fueled by open-source Earth Observations and social media information, it includes both physical and socio-economic characteristics of the shoreline. In a pilot study in the U.S. Virgin Islands (USVIs), most beaches were found to be vulnerable to erosion. The CVI was utilized to rank the most likely vulnerable beaches, which were then studied using historic geomorphologic data; these beaches were confirmed to be predominantly eroding. Significant erosion is projected as sea levels rise; for example, by 2050 under the RCP8.5 scenario, more than 50% of the 30 most vulnerable USVI beaches will erode by 50–100% of their current maximum width. The framework is designed to be used in vulnerable coastal settings that have limited financial and human resources.

**Supplementary Information:**

The online version contains supplementary material available at 10.1007/s13280-023-01946-w.

## Introduction

Sandy coasts (beaches) make up a significant portion of the global coastline (Luijendijk et al. 2018) and hold considerable hedonic and economic value (Boto-Garćıa and Leoni, 2022). They also offer protection from coastal flooding to the backshore natural and built environments, and their depletion can lead to substantial impacts (Jimenez et al. 2012; Pörtner et al. 2019; UNFCCC, 2020). On a global scale, many beaches are already under erosion (Mentaschi et al. 2018), which can be differentiated into (a) irreversible shoreline retreat due to relative mean sea level rise (RSLR) and/or negative sedimentary budgets that force landward migration and (potentially) drowning (Hinkel et al. 2013); or (b) short-term erosion caused by storm surges and waves. Beach erosion and its impacts are projected to accelerate under a changing climate due to RSLR and increasing intensity and/or frequency of storm surges/waves as well as continued human development of the coastal zone (e.g., Vousdoukas et al. 2020).

Beaches in island settings are particularly vulnerable because of their limited dimensions and sedimentary supply (e.g., Monioudi et al. 2023). As focal points of Sea-Sun-Sand (3S) tourism, many island beaches generate considerable economic activity that is vital to island economies. For example, 3S tourism-related economic activity accounts for at least 23% of the GDP of many Caribbean Small Island Developing States–SIDS (UNWTO 2019; Asariotis 2020; UNECLAC 2011). Given their heavy reliance on tourism, island economies can be severely impacted by beach erosion. If the goal is to maintain the appeal of 3S tourism destinations then management of beach erosion is critical (Rutty et al. 2020) particularly given the rapid changes occurring under climate change and variability. Unfortunately, the sandy beaches sought out by tourists are often the beaches most vulnerable to rising sea levels. For example, previous work suggests a relative sea level rise of just one meter would lead to partial or complete inundation for approximately 29% of resort properties across 19 Caribbean Community (CARICOM) countries (Scott et al. 2012). Compounding the challenge, the critical infrastructure that supports 3S tourism on many islands is also exposed to beach erosion and flooding as a result of RSLR and storms (Monioudi et al. 2018; Asariotis 2020; Bove et al. 2020).

Global and regional studies have brought attention to the escalating erosion risk faced by many sandy coastlines, including those in the Caribbean (e.g., Cambers 2009; Luijendijk et al. 2018; Vousdoukas et al. 2020). These studies primarily concentrate on identifying trends over large spatio-temporal scales, consequently, their practical relevance for managing local beach erosion remains limited to identifying general geographic areas of potential concern. Moreover, despite beach tourism significantly boosting the economic activity of 3S island destinations, the potential socio-economic consequences of erosion on tourism are usually not considered when planning for the future. For example, basic data such as tourist visits to individual beaches that could be used to help understand these consequences are frequently overlooked.

To address these gaps in beach erosion assessments, we have developed a framework for retrieving, compiling, and analyzing relevant information to systematically assess and rank the vulnerability of beaches at island level. Ranks are based on socio-economic importance, physical characteristics, and exposure and vulnerability to RSLR and extreme storm events. The system is piloted in The United States Virgin Islands (USVI), which are similar to many small islands in the Caribbean and elsewhere that rely on coastal development for income but face an urgent need to preserve and protect coastal resources from development and climate change. Although resources for coastal zone management in the USVI have historically been limited, datasets generated by U.S. national agencies (USGS, NOAA) are available for many areas in the islands. Similar to many other areas, the ongoing challenge is efficient (given continued limited resources) and effective use of resources for adaptation planning.

## Materials and methods

### Study area

The United States Virgin Islands (USVIs), a territory of the United States, (land area: 346.36 km^2^, population > 100,000) are located in the Northeastern Caribbean Sea, comprising the eastward extent of the Greater Antilles island arc system (Macdonald et al. 2000). The three Islands include St. Croix, St. John, and St. Thomas. St. Thomas and St. John are located in the Greater Antilles deformed belt (Gori and Hays 1984) and are subaerial topographic highs on the Puerto Rico Bank (Rankin 2002). St. Croix, located to the south, is situated in the Northern Caribbean deformed belt and is separated from the Puerto Rico-Virgin Island Platform by the Anegada trough, with depths exceeding 4,000 m (Case and Holcombe 1980a, b). International 3S tourism is vital for the economy of the USVIs, accounting for about 48% of total exports, 29% of the economy (2019), and 38% of (pre-pandemic) employment (WTTC 2021). Natural hazards, particularly hurricanes, have played a significant role in shaping the natural and human history/culture of the islands (Prevatt et al. 2010). Most recently, the 2017 hurricane season saw the devastating impacts of hurricanes Irma and Maria that caused damage to structures and infrastructure along the coastline, significant wind and rain damage inland, and severe coastal erosion from two storms making landfall two weeks from each other (Cox et al. 2019).

### Workflow

**Fig. 1:**
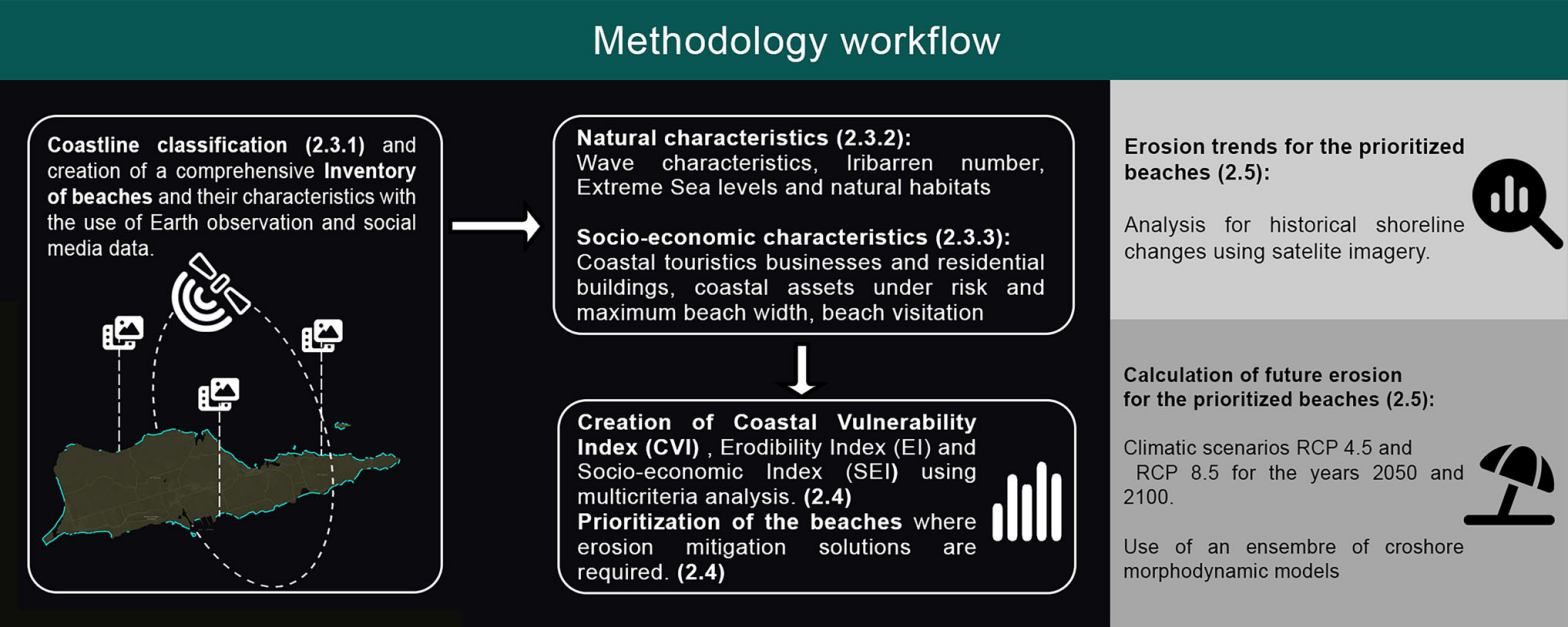
Graphical workflow of the method. The numbers refer to the manuscript sections

Details of the datasets and analytical methods are presented in following Sections "Beach Natural and Socio-economic environment," "Indices," and "Beach erosion trends and predictions." The development of the present framework (Coastal Information and Assessment System–CIAS, Fig. 1) commenced with the identification/classification of USVIs’ beaches (beach polygons) using Google Earth imagery. Other data were then collected and/or calculated based on the geographic location of beach polygons; details of the procedures are provided in subsequent sections. Briefly, environmental data includes waves (open source products of the Marine Copernicus Services Database), beach Iribarren numbers and natural habitats (Lidar topo-bathymetric and LandSat 8 imagery from the National Oceanic and Atmospheric Administration–NOAA), extreme Sea Levels (ESLs) (EC Joint Research Center JRC), and maximum beach widths (MBWs) (Google Earth). Data used for the assessment of economic or social/touristic importance include coastal residential development, coastal infrastructure and coastal businesses (variables retrieved from the Federal Emergency Management Agency–FEMA), and tourist visits (inferred from the annual average number of photographs taken in close proximity to each beach and subsequently uploaded to Flickr). Data were extracted from the respective datasets and linked to the beach polygon data.

The collated beach dataset was then used to estimate a Coastal Vulnerability Index (CVI) using the (Technique for Order of Preference by Similarity to Ideal Solution) multi-criteria analysis (TOPSIS) (Tzeng and Huang 2011). The CVI is used here to identify the “most vulnerable beaches” defined as beaches with high economic importance that are also the most prone to coastal erosion. Two additional indices (Erodibility and Socio-economic indices) were also created in order to expand the versatility of the framework. Finally, a subset of the most vulnerable beaches were further analyzed for historical shoreline changes and assessed for future shoreline erosion potential under different climate change scenarios using an ensemble of cross-shore morphodynamic models.

### Beach natural and socio-economic environment

#### Classification of the coastline

**Fig. 2:**
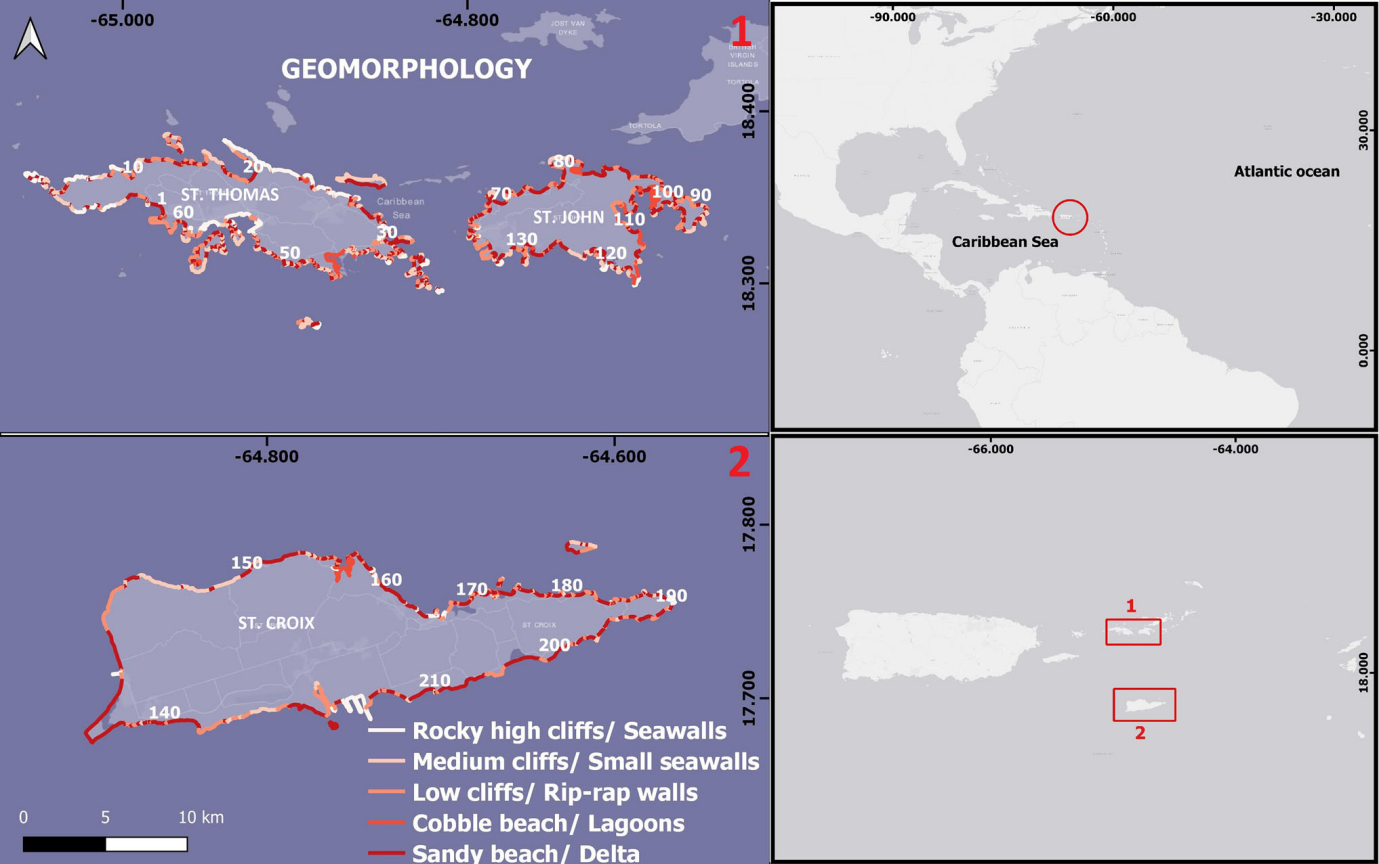
The Virgin Islands US Geomorphology map and beaches

A shoreline (line) file (.kml) was created using heads-up digitizing (Becker et al. 2021) in Google Earth Pro. The geomorphology of the study area was then roughly classified (for 100 m segments) into five categories: sand beach, cobble beach, low cliff/rip-rap walls, medium cliff/small seawalls, and rocky high cliff/seawalls based on the information presented in Hammar-Klose and Thieler (2001). Categorization of beaches and rigid structures (seawalls, rip-rap walls) was further refined from *in-situ* observations and a Digital Elevation Model.^1^ Two hundred and ten sandy segments (beaches) were identified for all of the USVI, 75 on St. Croix, 74 on St. John, and 61 on St. Thomas (Fig. 2). Polygons for beaches were then fashioned for each sandy beach segment identified using the most recent high-resolution satellite images from Google Earth collected within the same month for each beach between 2019 and 2022. Natural and socio-economic variables were then assigned to these polygons based on their geographic location. To approximate the carrying capacity of beaches, their total area was combined with the optimal number of users a beach can accommodate with comfort (22 m^2^/person, Chen and Teng (2016)) which yielded 48,100 individuals.

#### Natural environment

The Simulating Waves Nearshore (SWAN) spectral wave model was used to compute the spatial distribution of wave exposure. The model was run stationary for a one-year times-series for eight main directions and boundary forcing for 2020. The 90th percentile wave condition values for the dominant direction were derived from the Marine Copernicus Services Database^2^ and bathymetry data from GEBCO_2022 Grid.^3^ Each beach was given a wave energy value from the closest available model output grid points.

To assess the effects of beach typology on coastal retreat, the Iribarren number (ξ), ξ = β/(Ho/Lo)^1/2^) was calculated, where β is the beach slope and Ho and Lo the offshore wave height and wavelength. The Iribarren number (ξ) is a dimensionless parameter that describes some of the effects of waves on a beach. Lower values (< 0.5) represent dissipative beaches (milder slopes and/or increased wave steepness) that generally favor higher coastal retreat (e.g., Allenbach et al. 2015) and higher numbers more reflective surf zones. Iribarren numbers for beaches were based on slope derived as linear profiles (Monioudi et al. 2017) from 2019 Lidar topo-bathymetric dataset^4^ and wave height and period from the closest SWAN model output. Low Iribarren numbers typically indicate dissipative conditions and high numbers reflective conditions (Ruggiero et al. 2004; Allenbach et al. 2015). Projections of extreme sea levels (ESLs) were retrieved from the dataset described in Vousdoukas et al. (2018) and available on the EC-JRC website.^5^ These data use a baseline of the mean 1980–2014 period and extreme events with a return period of 100 years. Each beach was assigned the closest extreme sea level value.

In order to estimate the CVI, it is important to consider coastal habitats, which can play an important role in providing protection against beach erosion (e.g., Peduzzi et al. 2022). Benthic habitat polygons, which represent 26 different types of habitats, were obtained from NOAA (Kendall et al. 2001). The natural habitat index was developed using the InVest Coastal vulnerability supervisory model (Guerry and Ruckelshaus 2012) with ranges from 1 (high protection) to 5 (low protection) provided by each habitat type.

The maximum width for each beach was derived from polygons from the head-ups digitizing process from the high-resolution satellite images from Google Earth. Maximum beach width is important when assessing the vulnerability of a coastal region. A wide beach can act as a natural barrier that protects backshore environments from storm surges/waves as well as relative sea level rise (Landry and Hindsley 2011). A wide beach can also provide both long- and short-term coastal protection by reducing the risk of flooding and destruction of coastal infrastructure/assets.

#### Socio-economic environment

Several variables were taken into account to capture the socio-economic value of beaches. Data pertaining to coastal tourist businesses and residential buildings were derived from a dataset of building polygons provided by the Federal Emergency Management Agency (FEMA 2019). These building polygons were selected with the application of a 1 km buffer around each beach. It is worth noting that coastal communities might have digitized building information for zoning and tax purposes. In cases where such data are unavailable, a 'heads-up' digitizing process can be employed using Google Earth Pro, as outlined by Becker et al. (2021) and Bove et al. (2020). Coastal business and residential buildings were used to represent the count of buildings most likely associated with beach recreational activities. To further quantify the potential impact of severe coastal flooding events, an additional variable ('assets') was generated. This variable aimed to account for the number of buildings in the backshore zone likely exposed to extreme sea levels. To accomplish this, the total count of buildings situated within 100 m of the beach backshore area was included in the analysis. Typically, buildings within this zone could be susceptible to flooding, making their inclusion important for accurately estimating coastal vulnerability.

Despite our efforts, we encountered challenges in locating sources that routinely gather data on beach visitation, which are useful for the comprehensive socio-economic assessment of beaches. In light of this, the Visitation and Tourism component of the Invest model (Guerry and Ruckelshaus 2012) was employed. This model allowed us to derive visitation values based on the average annual number of photographs taken within the vicinity of each beach and subsequently uploaded to the Flickr database (Wood et al. 2013) over the period from 2005 to 2017. The visitation variable encompasses a 1 km radius around the centroid of each beach and serves as a proxy measure for the level of tourism or interest in that area.

### Indices

The Coastal Vulnerability Index (CVI) is a valuable tool used to assess the vulnerability of coastal areas to climate change, particularly in relation to the effects of sea level rise and extreme events. It includes combinations of physical characteristics of the shoreline, natural habitats, and human activities to provide a broad understanding of coastal vulnerability. The CVI has been widely used in research and planning in numerous coastal regions around the world (e.g., Gornitz 1991; Shaw et al. 1998; López Royo et al. 2016). The CVI proposed in this work is based on an unweighted TOPSIS multi-criteria analysis (Tzeng and Huang 2011). Unweighted TOPSIS was chosen as a simple, unbiased means to include both natural environment and socio-economic data that can be tailored to provide a wide range of possible alternatives. For example, in future use, variables can be weighted to reflect decision makers’ and/or study objectives. For each variable, the performance value for each beach was divided by the rooted summation of the square value in order to normalize the evaluation matrix. The ‘best’ and ‘worst’ values were found for each variable, and the Euclidean distance from both was calculated for each beach and performance scores summed for each beach and variable. Ten beaches with the highest CVI (indicating most vulnerable) on each of the three islands (30 total) were chosen for further assessment of long-term erosion trends and future beach retreats under climate change.

The Erosion (EI) and Socio-Economic (SEI) indices are also based on unweighted TOPSIS multi-criteria analysis (e.g., Andreadis et al. 2021). The EI quantifies erosion potential based on wave exposure, the presence/distribution of (beach protecting) natural habitats, extreme sea levels, and the Iribarren numbers. The SEI is calculated using the number of individual beach visits, coastal touristic businesses, residential development, coastal asset values, and maximum width to account for backshore protection. The EI and SEI were not used to prioritize beaches for further analyses, but as a visualization tool to present a clearer narrative of the study area. The EI and SEI can help highlight beaches that have a low CVI values that are “important” on the basis of socio-economic and/or natural characteristics.

### Beach erosion trends and predictions

The Digital Shoreline Analysis System (DSAS) (Hapke et al. 2011; Himmelstoss et al. 2018; Thieler et al. 2009) was used for analyzing changes to the historical coastline position. DSAS was run for the 30 prioritized/highest scoring beaches (see above) in the three USVIs for the years 2002–2021. The number of images analyzed for each beach (500 total for 30 beaches) depended on image availability in Google Earth Pro (GEP). Shoreline position was created using heads-up digitizing of the sand/water interface in GEP images of 1 m (or higher) resolution. The location of the beach land–water interface is highly dynamic on both short and long time scales and this may introduce errors when analyzing long-term changes in shoreline position. To account for some of the possible seasonal uncertainty, images were collected within ± 2 months for each beach for each year analyzed.

To efficiently analyze hundreds of images, a methodology was needed to control image alignment and sensor angle without requiring the export and geo-referencing of images prior to shoreline delineation. First, images were adjusted to an altitude of 300 m elevation with zero tilt in Google Earth Pro following Warnasuriya et al. (2020). The shorelines were then digitized, and ground control points (GCP) were placed on prominent features in the most recent image for each beach. Measurements (using GEP ruler) were taken from the GCP position to identical ground positions in subsequent images for which the shorelines were recorded. The “error” or shift measurements were then recorded in the DSAS database for each shoreline as “shoreline positional uncertainty” measures, a pre-programmed feature in DSAS (Himmelstoss et al. 2018).

The coastline response to the long-term sea level rise was projected using an ensemble of cross-shore (1-D) analytical morphodynamic models, i.e., the Edelman (1968), Bruun (1988), and Dean (1991), models (Monioudi et al. 2017). The models were set up and forced on the basis of the observed cross-shore natural profiles derived from the 2019 topo-bathymetric Lidar data and the energetic wave characteristics calculated from the results of the SWAN model.

RSLR projections along the coasts of U.S. Virgin Islands were retrieved from the EC-JRC (Joint Research Centre) database (Vousdoukas et al. 2018). The scenarios considered were RCP4.5 and RCP8.5 for the years 2050 (SLR 0.26 m and 0.32 m, respectively) and 2100 (SLR 0.57 m and 0.9 m, respectively). These Representative Concentration Pathway scenarios were considered to project the impacts on the USVI beaches under what are considered moderate (RCP 4.5) and high emissions (RCP 8.5) scenarios.

## Results

### Coastal vulnerability index variables: Spatial distribution

#### Wave exposure and Iribarren number

**Fig. 3:**
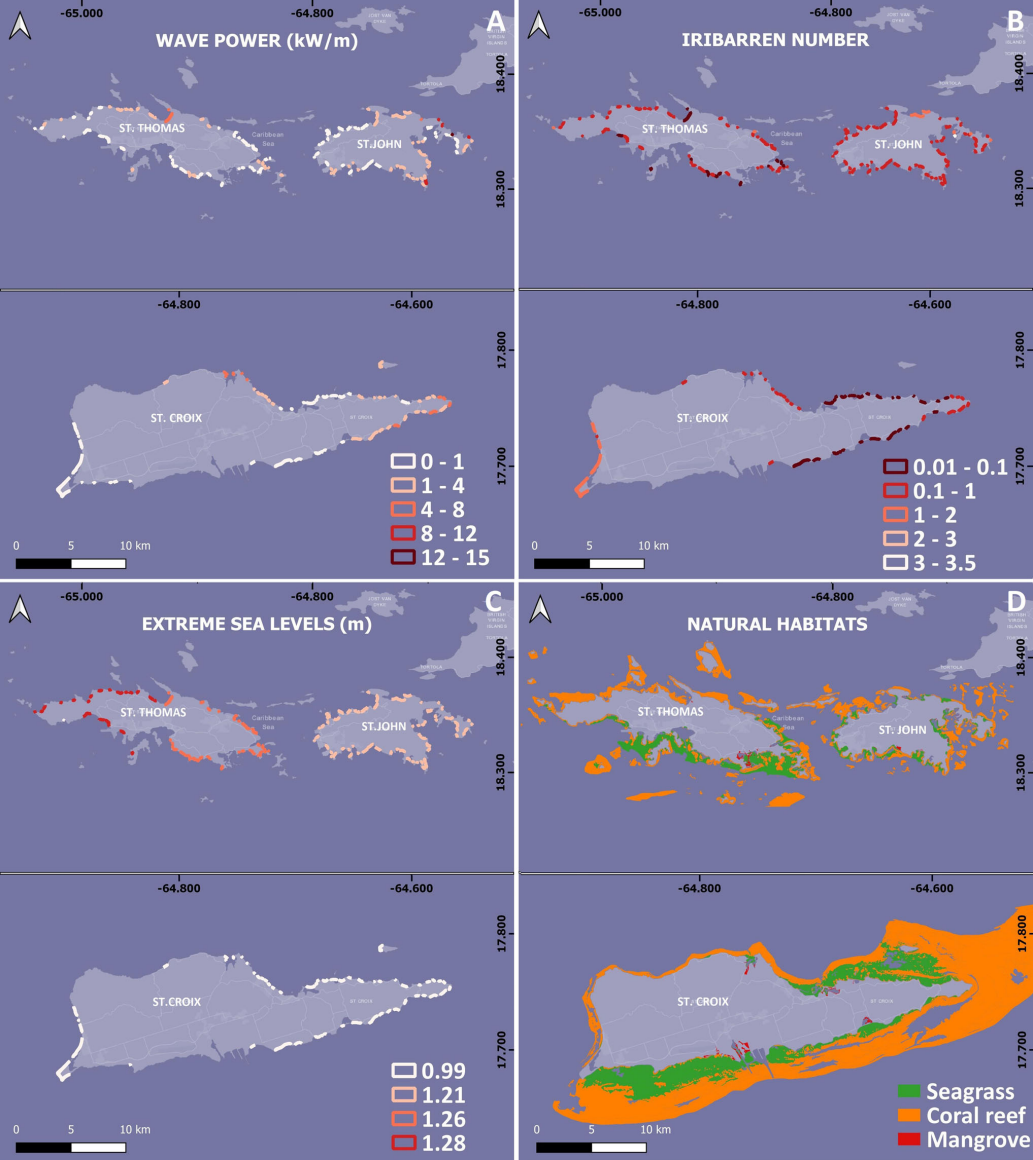
Distribution of the selected variables for the Coastal Vulnerability Index calculation along the beaches of the US Virgin Islands: **A** Wave Power: **B** Iribarren number; **C** extreme sea levels; and **D** natural habitats

In general, the spatial distribution of exposure to waves (Fig. 3A) shows a higher exposure for eastward facing beaches with long fetch distances. Prevailing waves are from the east for all islands. The peak 90th percentile wave power is over 10 kw/m at the eastern coasts of St. John and St. Croix. St. Thomas is more protected from eastern waves and, thus, wave power is low along its eastern and western coasts with peak values found along its northern shoreline (up to 5 kw/m). Minimal wave power values are also found along the western St John coast, and the western, southern, and northern coasts of St. Croix.

Across the majority of the beaches, the Iribarren numbers exhibited low values, which implies that these beaches exhibit dissipative traits that contribute to coastal retreat. The range of values varied from nearly zero (beaches with a juxtaposition of elevated mean wave heights and exceedingly gradual slopes) to almost 1 for beaches characterized by a combination of lower mean wave heights and steeper inclines (Fig. 3B).

#### Extreme sea levels and coastal habitats

Extreme Sea Levels (ESLs) for a return period of 100 years had maximum values for the baseline period 1980–2014 of 1.28 m and 1.21 m along the western and eastern coasts of St. Thomas, respectively. Similar ESL’s (1.19 m) were estimated for the coast of St John with the lowest ESL values (0.99 m) on the coast of St. Croix (Fig. 3C). Natural ecosystems that can positively influence beach resilience, such as nearshore coral reefs and seagrasses (e.g., Peduzzi et al. 2022), are abundant along the USVI coast. The coastline of the USVI is largely fronted by coral reefs, with the exception of the southwestern coast of St. Thomas, small areas of the southwestern and northern coast of St. John and the western coast of St. Croix. Seagrasses appear along the southern, southeastern, and eastern coasts of St. Thomas, along the shore of almost the entire coast of St. John, and on the northeastern and southern coasts of St. Croix. Mangroves, which also provide protection to the coast, can be found in small areas of all three islands (Fig. 3D).

#### Tourism, visitation, and residential development

**Fig. 4:**
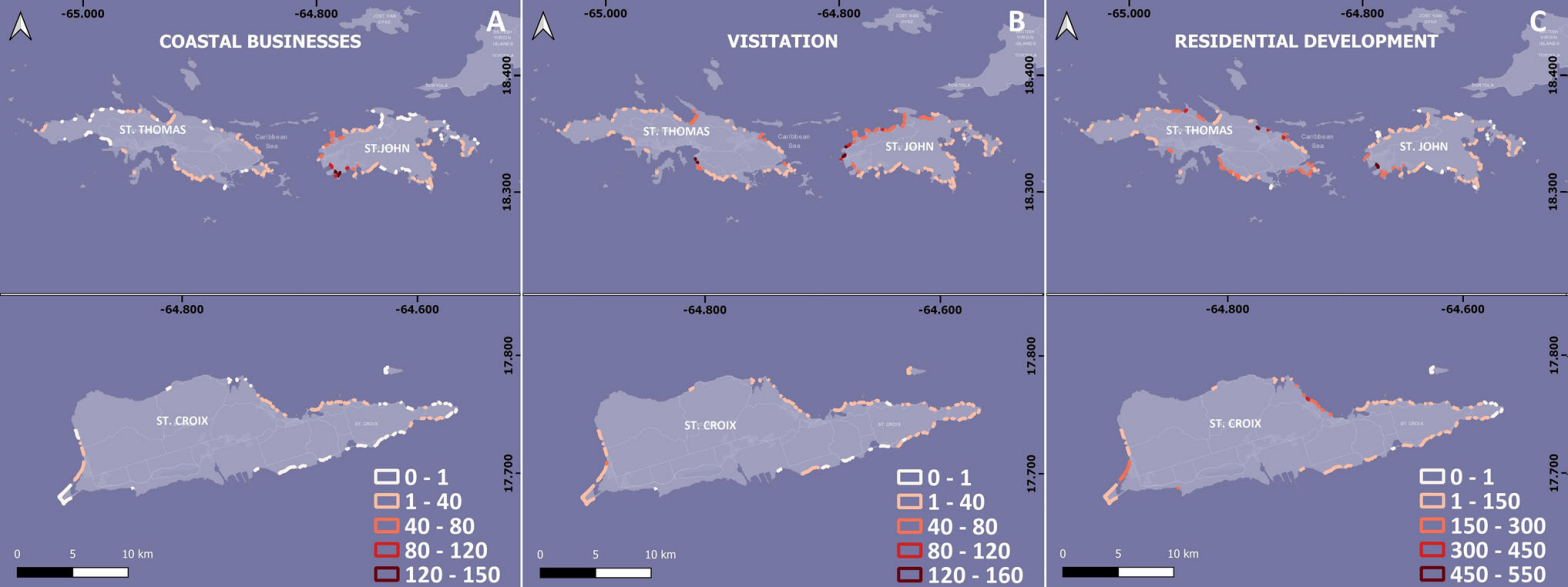
Distribution of the selected variables for the Coastal Vulnerability Index along the beaches of the US Virgin Islands: **A** Number of coastal businesses in a 1 km radius; **B** Visitation (number of photos uploaded); and **C** Residential development (number of residential buildings in a 1 km radius)

The northwestern and southwestern coasts of St. John have the highest concentration of hotels and tourism-related businesses, as well as uploaded photos in the Flickr database (Fig. 4A, B), indicating they are likely the most popular tourist destinations, particularly its southwestern beaches. Although St. Thomas and St. Croix appear to have fewer visitors overall, several beaches on the western and northern coasts appear to receive a significant number of visits (Fig. 4B). Concerning local communities, St. Thomas has the highest population density and the greatest number of residential buildings near beaches, whereas St. John's residential development is mainly concentrated along the southwestern and western coasts, due to the presence of the proportionally large national park on the island. St. Croix's residential development is concentrated on the northern and western coasts (Fig. 4C). Generally, the lowest densities of residential development are found in the eastern areas of St. John and St. Croix and western St. Thomas (Fig. 4C). While perhaps not surprising, it is of interest to note that in general, the spatial distribution of the socio-economic variables used in the analysis (businesses and hotels, visits, residential development, number of assets/buildings) is inversely proportional to wave exposure: areas with smaller wave exposure have higher numbers of buildings, more visits, and overall development. Examples include eastern St. Thomas, western St. John, and the western and northern areas of St. Croix.

#### Assets and maximum beach width

**Fig. 5:**
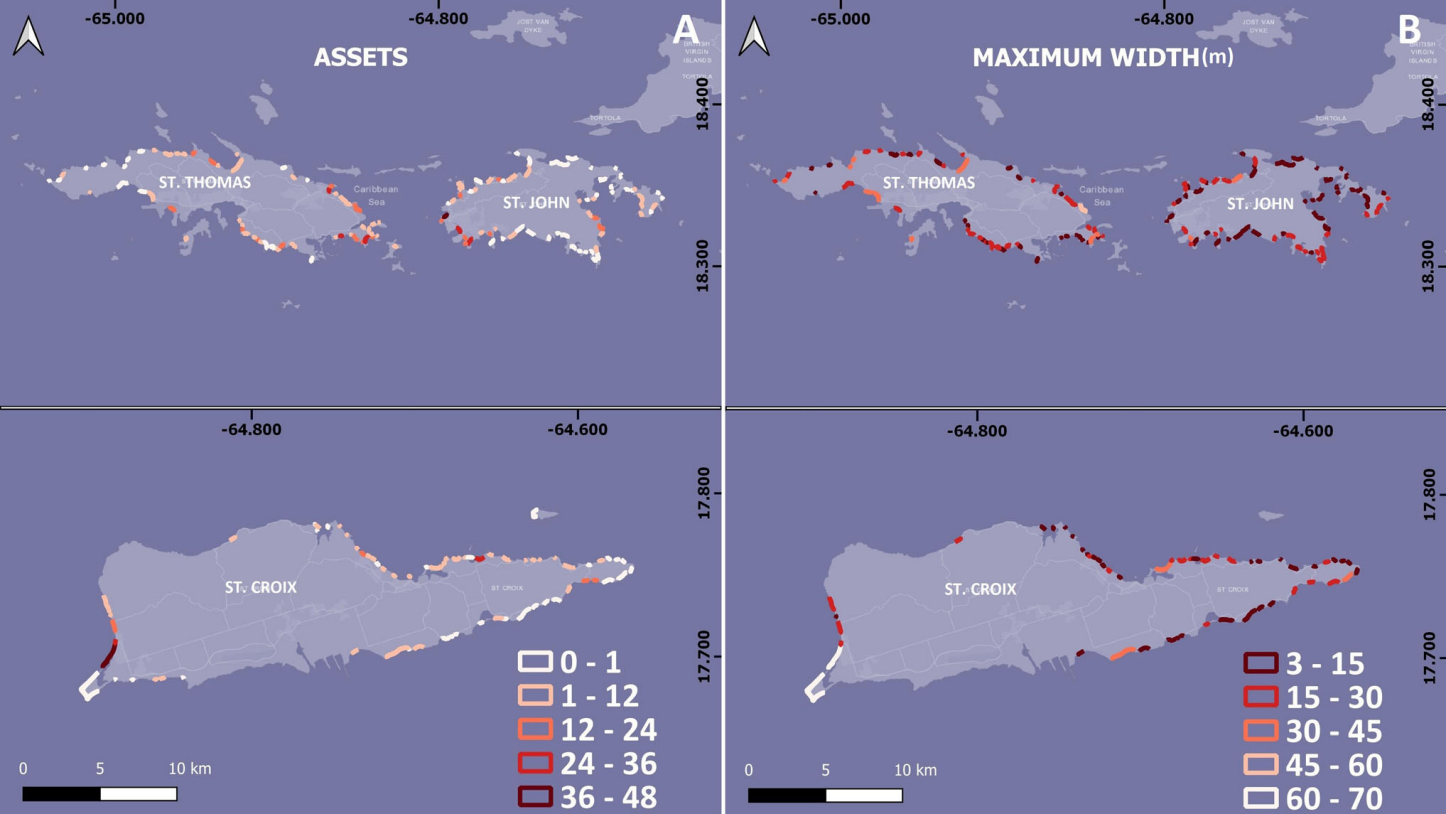
Distribution of the selected variables for the Coastal Vulnerability Index calculation along the beaches of the US Virgin Islands: **A** Assets (number of backshore buildings in a 100 m radius); and **B** Maximum beach width

The distribution of coastal assets closely mirrors that of residential development, with the greatest number of assets found near the beaches of eastern St. Thomas and western St. John. Coastal assets in St. Croix are concentrated along the northern and western coasts (Fig. 5A). The eastern coasts of St. John and St. Croix, as well as the western coast of St. Thomas have the lowest number of assets (Fig. 5A). Concerning beach width, the average maximum width of all beaches is approximately 14 m, and most beaches are narrow with small maximum widths (less than 20 m) and high maximum width indexes. In combination with low Iribarren numbers, this suggests that most beaches on the islands are vulnerable to erosion and likely do not provide significant protection to the backshore (Fig. 5B). A concerning observation pertains to numerous beaches along the eastern coast of St. Thomas and the western coast of St. John. These beaches exhibit a convergence of numerous assets in their backshore areas with low Iribarren numbers, notably small maximum widths (most measuring less than 30 m, and in some instances less than 10 m) (Fig. 5B) and, for St. Thomas higher extreme sea levels. This points to the potential for this framework to locate potentially vulnerable or exposed infrastructure and socio-economic activities.

### Indexes, spatial distribution, and beach prioritization

**Fig. 6:**
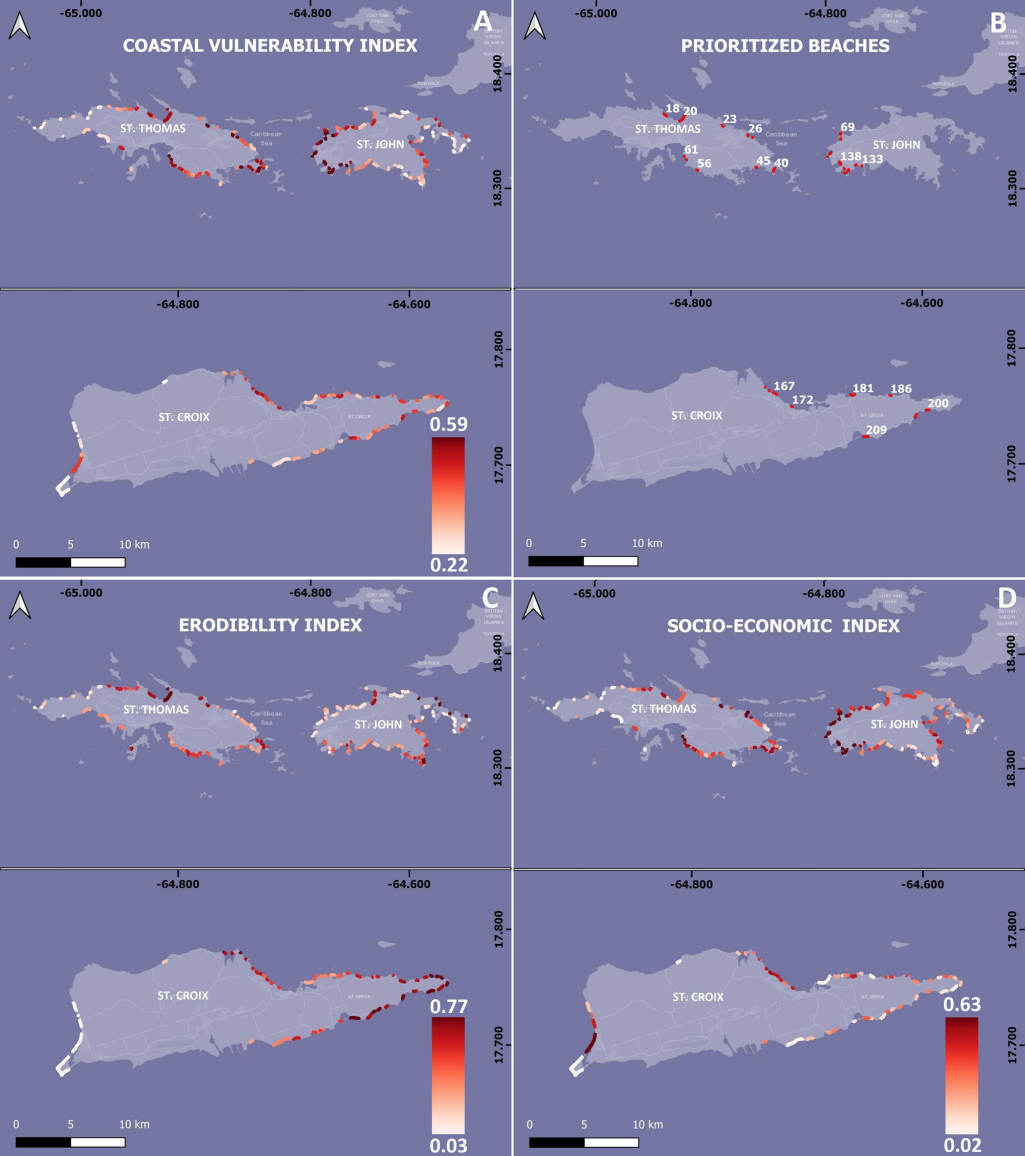
**A** Spatial distribution of the Coastal Vulnerability Index (CVI) (0, least vulnerable—1, most vulnerable) for beaches of the US Virgin Islands. **B** Highest 10 CVI values of each island’s beaches. **C** Spatial Distribution of the Erodibility Index (EI) (0, least prone to erosion–1, most prone to erosion) and **D** Spatial Distribution of the Socio-Economic Index (SEI) (0, less important—1, most important)

The Coastal Vulnerability Index (CVI) provides a ranking of beaches based on their vulnerability and socio-economic importance with a range of 0 (least vulnerable) to 1 (most vulnerable); USVI beaches range from 0.22 to 0.59. The CVI shows (shown in Fig. 6A) the largest number of vulnerable beaches are located on St. Thomas and St. John, with the most vulnerable individual beaches on the western coast of St. John. St. Croix has the lowest range of CVI values and the least vulnerable beaches in the islands (Fig. 6A). The ten beaches with the highest CVI for each island were identified as being at the highest risk and selected for the next steps of the study (see Table 1, Fig. 6B).

**Table 1:** Prioritized beaches for each island based on CVI ranks

St. Thomas	CVI rank	ID	St. John	CVI rank	ID	St. Croix	CVI rank	ID
Water Bay	1	26	Great Cruz Bay	1	135	Unknown Beach 38	1	159
Prince Ruperts Cove	2	59	Hult Bay	2	132	Unknown Beach 40	2	162
Unknown Beach 19	3	58	Galge Cove	3	63	Grapetree	3	194
Scott Beach 2	4	43	Chocolate Hole	4	134	Danish building	4	161
Sugar Bay	5	27	Monte Bay	5	131	Solitude Bay	5	180
Tutu	6	23	Caneel Bay	6	66	Christiansen Harbor	6	166
Cowpet Bay	7	38	Frank Bay	7	62	Chanay	7	175
Unknown Beach west of Maggens Bay	8	18	Unknown Beach 32	8	133	Pelican Cove	8	157
Unknown Beach 18	9	54	Turtle	9	67	Devi	9	195
Maggens Bay	10	20	Monte Bay 2	10	130	East end marine park	10	202

The Erodibility Index (Fig. 6C) ranks beaches based on their vulnerability to erosion. Index values range from 0 (least vulnerable) to 1 (most vulnerable); USVI beaches range from 0.03 to 0.77. The highest values for erodibility are along much of the coast of St. Thomas, southern and eastern St. John, and the eastern and northern coasts of St. Croix. Lower values are estimated for the northwestern coast of St. Thomas, northwestern St. John, and a few beaches in the eastern and western areas of St. Croix.

The Socio-economic Index (Fig. 6D) ranks beaches based on their economic importance, with values ranging from 0 (less important) to 1 (most important); USVI values range from 0.02 to 0.63. The highest values were estimated for St. John, particularly its western beaches, as well as in St. Thomas and the western coast of St. Croix. The lowest SEI values were estimated for some of the most exposed beaches on the eastern coast of St. John, western St. Thomas, and parts of the southern and southeastern St. Croix coasts.

### Beach erosion trends and Digital Shoreline Analyses Software results

The prioritized beaches for each island were further assessed for long-term erosion trends using the Digital Shoreline Analyses Software (DSAS); results are given in Table S1). The DSAS results are based on transects cast at 10 m intervals perpendicular to and through all digital historic shoreline positions from a fixed baseline created onshore set back from each beach ~ 50 m. The results are predominated by erosion with very few instances of long-term accretion. On St. Croix out of a total of 480 transects analyzed, 393 (82%) were found to be erosional and 87 (18%) accretional; moreover, 186 (47%) of the erosional and only 5 (6%) of the accretional transects were found to be statistically significant (90th percentile). On St. John, there was a total of 213 transects on 10 beaches; 129 (61%) were erosional and 84 accretional, 18% and 5% of the erosional and accretional transects, respectively, were statistically significant. For St. Thomas, 275 (88%) of a total 330 transects for the ten prioritized beaches were erosional and 37 (12%) accretional; of the 275 erosional transects, 139 (51%) were statistically significant for erosion and < 1% of the 37 accretional transects for accretion (90th percentile). Although some beaches exhibited a mix of erosion and accretion, in all cases the number of transects that exhibited statistically significant erosion outnumbered the transects showing beach growth by more than a 2:1 ratio. Finally, the greatest average significant rate of loss of beach width was estimated as 46 cm/year on St Croix, 42 cm/year on St John (STJ), and 56 cm/year on St Thomas (STT) (Table S1).

### Future beach retreat scenarios

The response of the 30 prioritized beaches to long-term SLR (Table 1) was projected using an ensemble of three cross-shore (1-D) analytical (Edelman 1968; Bruun 1988; Dean 1991) morphodynamic models. The scenarios used for sea level rise were RCP 4.5 and RCP 8.5 for the year 2050 (SLR 0.26 m and 0.32 m, respectively) and RCP 4.5 and RCP 8.5 for the year 2100 (SLR 0.57 m and 0.9 m, respectively). Model outputs show that many of the prioritized beaches in the USVIs appear to be under serious threat of future erosion due to sea level rise, with beach retreat estimated between 1.5 m and 27 m by the year 2050 under the more conservative RCP 4.5. In more detail, the model indicates a 75–100% reduction in maximum width for nine of the 30 beaches, a reduction of 50–75% for seven more, and a 25–50% reduction for one. Under the more extreme RCP 8.5 scenario, greater erosion was estimated: with beach retreat between 2 and 30 m leading to a 75–100% reduction in maximum width for 13 beaches, 50–75% reduction for three beaches, and 25–50% reduction for three more of the 30 prioritized beaches.

For the year 2100 for RCP 4.5, beach retreat was estimated between 3 and 50 m with more than half (16) of the beaches projected to be reduced by 75–100% of their current maximum width, one reduced by 50–75%, and six reduced 25–50% of their current maximum width. Finally, for the most extreme time and SLR scenario modeled (year 2100, RCP 8.5), beach retreat of between 4 and 85 m was estimated, with dire consequences for the majority of the prioritized beaches. In this scenario, 17 beaches disappear completely, four are reduced by 50–75% and seven reduced by 25–50% of the year 2020 measured maximum width (Table S2).

## Discussion and conclusions

Using the Coastal Vulnerability Index (CVI), created on the basis of both physical and socio-economic characteristics, the USVI’s beaches were ranked according to vulnerability. The index facilitated (by ranking) the identification of 10 beaches on each of the three US Virgin Islands that have high socio-economic value and are prone to erosion. These most vulnerable beaches were verified to be already under erosion based on the DSAS results and are also likely to face large beach retreats in the future under sea level rise based on the results of the 1D models’ ensemble. The highest erosion rates were found for the beaches of St. Thomas and St. Croix with lower rates found for St. John. Results for future beach retreat roughly follow past and current erosion trends found in DSAS, with six out of ten beaches of St. Thomas and seven out of ten of St. Croix projected to completely disappear by 2100 under a RCP 8.5 scenario, and two out of ten disappearing in St. John for the same scenario.

In general, there was a high level of agreement between analyzed past erosion and predicted future erosion trends for both extreme and moderate values which adds validity to the methodologies used to identify beach vulnerability under sea level rise. For example, in St. Thomas, Sugar Bay beach was found to have very low past/current erosion rates of approximately 0.05 m per year, and results for its future retreat values range on the lower end of all beaches from 2.5 m for the RCP 4.5 scenario in the year 2050 to 8.2 m for a RCP 8.5 scenario in the year 2100. Water Bay beach on the other hand shows a higher current erosion rate (approximately 0.56 m per year) and very high projections for future retreat, ranging from 13.4 m for the RCP 4.5 scenario in 2050 to 43 m for the RCP 8.5 scenario in 2100. There are similar results for the beaches of St. John, where lower past/current erosion rates are associated with low (likely) future retreat (Frank Bay Beach, Galge Cove Beach, Turtle Beach, Monte Bay 1 and 2 beaches), and higher past/current erosion rates correspond to higher future retreat (e.g., Great Cruz Bay Beach). In St. Croix, while there are examples that show a similar pattern (e.g., low values both in past/current erosion and future retreat for Solitude Bay Beach and high values in Grapetree Beach), there are also inconsistencies between the current erosion and future beach retreat. This was found for certain prioritized beaches located in the northern part of the island near Christiansted Harbor (Pelican Cove, Unknown Beach 38, the Danish Building, and Unknown Beach 40, see Table S2), as well as Great Pond Bay (East End Marine Park) in the southeast. Although DSAS results show low erosion rates for these beaches, the morphodynamic model ensemble predicts likely high future beach retreats. These inconsistencies might be due to limitations of the 1-D analytical morphodynamic models which do not account for factors that might limit erosion such as artificial beach protection schemes or the protection afforded by nearshore ecosystems (Monioudi et al. 2017). We note specifically that these beaches are fronted by large coral reefs (e.g., Long Reef in northern St Croix) which may act as submerged breakwaters and also supply the beaches with biogenic sediments (Peduzzi et al. 2022). These results should be considered in the context of the future of coral reefs in the USVI’s and across the Caribbean which face serious local (Ennis et al. 2016; Edmunds et al. 2019; Brandt et al. 2021) and global threats (Cramer et al. 2021; Munoz-Castillo et al. 2019). In a scenario of diminished coral reef health, although sedimentation supply might increase in the short term, the projection that reefs afford beaches would likely be reduced. Another modeling limitation is that the projections rely on beaches composed of infinite sediment reserves that do not experience horizontal sediment losses; a limitation cannot be addressed in cross-shore modeling (Monioudi et al. 2017, 2023). Furthermore, an additional concern arises regarding the prioritization of the Coastal Vulnerability Index (CVI) in this study. While the CVI takes into consideration the protection of coastal habitats through the natural habitats index (Guerry and Ruckelshaus 2012), it lacks detailed categorization that adequately accounts for the extent and density of these habitats. In order to improve the accuracy of future retreat modeling and to better address the protection provided by submerged reefs and other habitats, it is recommended that future studies incorporate more of these details into the protocols. This can be achieved by developing a new natural habitats index that incorporates more detail of the extent and density of coastal habitats along with the possible inclusion of a ‘coastal protection index’ designed for beaches protected by submerged coral reefs and rigid structures. By implementing these enhancements, a more comprehensive assessment of coastal vulnerability can be achieved, ultimately informing more effective coastal management strategies.

Recent studies have demonstrated the socio-economic significance of beach carrying capacity and its dynamics. For example, Rodella et at. (2020) found when using a willingness to pay (WTP) approach, the non-market value of the sustainable carrying capacity in selected Italian beaches varied from more than €50 million per season at a popular urban beach to €1 million at a remote natural beach and concluded the huge non-market value of these beaches should provide incentives for decision makers to pursue beach protection and restoration measures. In the current study, using a simplified method based on the optimal beach carrying capacity (number of individual users a beach can accommodate) of 22 m^2^/person (Chen and Teng 2016), the prioritized beaches can currently accommodate around 6,100 individuals. Considering only those beaches that experience the largest impact (those that will completely disappear due to erosion rather than the majority that will see partial retreats), total carrying capacity is projected to decline by 2050 to approximately 5,500 and 5,300 for RCP 4.5 and RCP 8.5 scenarios, respectively. By 2100, the carrying capacity is expected to further decrease to around 4,600 and 4,500 for the same scenarios. Many of the beaches that are projected to have the largest impact are also the most popular tourist destinations based on visitation, hotel numbers, and residential development included in the CVI. Even with this conservative (considering only beaches projected to be completely lost) estimation, the economic repercussions of lost carrying capacity could be catastrophic for an area heavily reliant on the economic activity that tourist visits generate.

This study lays the foundation for a Coastal Information and Assessment System (CIAS) that can be developed with publicly available resources. This system can help decision makers identify beaches that have undergone the highest rates of erosion, predict future erosion trends, and understand where future income from beach tourism might be lost as a result of climate change. These are pragmatic first steps in planning for future climate change scenarios through understanding potential losses based on the weighting of input information that includes both socio-economic and environmental data. Data selection in the current analyses reflects an interest in demonstrating the open source nature of the framework, but the system is flexible and a wide range of inputs are possible depending on data availability and user interests. For example, while high-resolution data (e.g., LIDAR topo-bathymetric data from NOAA) can improve accuracy (e.g., for the Iribarren number and morphodynamic modeling), the methodology can be also applied in any setting globally (albeit with lower accuracy) using open access Digital Elevation models, such as SRTM or EU-DEM and satellite derived bathymetry with Sentinel 2 satellite images (Caballero et al. 2019). These have shown to be reasonable substitutions when higher resolution DEM’s are unavailable (Bove et al. 2020). Other data can also be approximated or estimated as demonstrated by the use of photos, which in the current work were used to determine beach visits. Potential users are encouraged to understand that inputs can be estimated using techniques that allow for useful assessments even if “optimal” data are not available.

Finally, beaches play important roles in the culture, ecology, and economies of small islands around the globe, but the threat of CC&V to their existence does not often receive the attention that other coastal systems (transportation, utilities, etc.) that are also at risk from climate change (e.g., Monioudi et al. 2018) do. As communities and governments struggle to assess and plan for the impacts of climate change in the coastal zone, the single most important income generator and cultural asset for many Caribbean 3S destinations should be part of those plans. Results in the current work point to an urgent need for careful planning, or the economic impacts of erosion on island economies could be severe as soon as the year 2050. The flexible system presented here leverages open access data and can provide a base for future work to incorporate a variety of measures related to the coastline. Inputs that highlight unique characteristics of segments of the coast and include local knowledge and economic impacts important for planning responses to climate change should be part of any plan for resilience.

### Supplementary Information

Below is the link to the electronic supplementary material.

Appendix. Supplementary file1 (PDF 88 KB)

## Footnotes

1 https://coast.noaa.gov/digitalcoast/data/home.html.

2 https://data.marine.copernicus.eu/product/GLOBAL_ANALYSIS_FORECAST_WAV_001_027/description.

3 https://download.gebco.net/.

4 https://coast.noaa.gov/dataviewer/

5 http://data.jrc.ec.europa.eu/collection/LISCOAST.
