# Supplementary material for: A system for the management of sandy shorelines under climate change: United States Virgin Islands (USVI)
Source: Ambio. 2023 Nov 30;53(3):406–20. doi: 10.1007/s13280-023-01946-w (PMC10837408; doi:10.1007/s13280-023-01946-w)
Supplement: Supplementary file 1 — Appendix. Supplementary file1 (PDF 88 KB) [file 13280_2023_1946_MOESM1_ESM.pdf]

Ambio

Electronic Supplementary information
This supplementary material has not been peer reviewed.

Title: **A SYSTEM FOR THE MANAGEMENT OF SANDY SHORELINES UNDER CLIMATE CHANGE: UNITED STATES VIRGIN ISLANDS (USVI)**

Authors: Theodoros Chalazas, Gerald Bove, Dimitrios Chatzistratis, Isavela N. Monioudi, Adonis F. Velegrakis

Table S1: DSAS results for the prioritized beaches

Island	Beach name	Maximum Width (m)	ave rate Δ w/reduced uncertainty (+/-)	# of erosional transects	max Δ on a single transect	%stat sig. transects (90%)	ave Δ erosion rate all transects	# accrectional trans	max Δ on single transect	%stat sig. accrectional transects (90%)	ave rate of accretion
St Thomas	Unknown Beach west of Maggens Bay	6	-0.39 (0.09)	43	-0.71	95	-0.39	0	-	-	-
St Thomas	Maggens Bay	33	-0.15(0.15)	92	-0.39	8	-0.2	19	0.16	0	0.16
St Thomas	Tutu	7	-0.35(0.12)	17	-0.89	71	-0.48	4	0.38	14	0.21
St Thomas	Water Bay	18	-0.56(0.14)	22	-1	95	-0.56	0	0	0	0
St Thomas	Sugar Bay	6	-0.05(0.04)	9	-0.06	22	-0.05	0	0	0	0
St Thomas	Cowpet Bay	40	-0.23(0.1)	45	-0.74	58	-0.3	7	0.44	4	0.26
St Thomas	Scott Beach 2	7	-0.23 (0.09)	16	-0.62	67	-0.26	2	0.01	0	0.01
St Thomas	Unknown Beach 18	7	-0.48(0.14)	17	-0.24	76	-0.24	0	0	0	0
St Thomas	Unknown Beach 19	15	0.13 (0.67)	5	-0.19	0	-0.11	5	1.28	0	0.36
St Thomas	Prince Ruperts Cove	9	-0.45(0.26)	9	-1.23	67	-0.45	0	0	0	0
St John	Frank Bay	10	-0.02(0.3)	4	-0.11	0	-0.07	4	0.08	0	0.03
St John	Galge Cove	14	0.1(0.24)	6	-0.33	0	-0.09	21	0.33	0	0.15
St John	Caneel Bay	10	0.01(0.2)	4	-0.35	0	-0.24	17	0.16	0	0.07
St John	Turtle	17	0.01 (0.2)	4	-0.35	0	-0.24	17	0.16	0	0.07
St John	Monte Bay 2	18	-0.01(0.24)	8	-0.29	0	-0.15	5	0.42	0	0.22
St John	Monte Bay	7	-0.07(0.08)	12	-0.29	35	-0.15	5	0.2	12	0.13
St John	Hult Bay	10	-0.03	24	-0.2	6	-0.07	11	0.16	31	0.06
St John	Unknown Beach 32	19	-0.42(0.15)	10	-0.42	100	-0.31	0	0	0	0
St John	Chocolate Hole	32	-0.05(0.26)	14	-0.18	0	-0.09	4	0.35	22	0.11
St John	Great Cruz Bay	17	-0.19(0.1)	43	-0.56	19	-0.19	0	0	0	0
St Croix	Pelican Cove	6	-0.26(0.17)	24	-0.55	21	-0.26	0	0	0	0
St Croix	Unknown Beach 38	8	0.01	11	-0.14	5	-0.07	8	0.28	0	0.13
St Croix	Danish building	8	-0.15(0.16)	18	-0.26	5	-0.16	1	0	0	0
St Croix	Unknown Beach 40	3	0.05(0.16)	3	-0.55	6	-0.35	13	0.23	6	0.14
St Croix	Christiansen Harbor	5	-0.46(0.13)	63	-0.99	67	-0.52	7	0.17	4	0.09
St Croix	Chanay	26	-0.22(0.16)	46	-0.62	38	-0.28	6	0.57	0	0.28
St Croix	Solitude Bay	5	-0.03(0.29)	15	-0.16	0	-0.06	6	0.04	0	0
St Croix	Grapetree	22	-0.4(0.12)	34	-0.71	71	-0.4	0	0	0	0
St Croix	Devi	20	-0.41(0.06)	152	-2.11	63	-0.5	19	0.74	8	0.33
St Croix	East end marine park	4	0.05(0.16)	27	-0.28	0	-0.12	27	0.8	9	0.23

Table S2: Static models long-term erosion results for the prioritized beaches for the 2050 RCP 4.5, 2050 RCP 8.5, 2100 RCP 4.5 and 2100 RCP 8.5 climatic scenarios.

INPUTS			STATIC MODELS LONG-TERM EROSION (ONLY MSLR FROM BASELINE FOR 4 SCENARIOS)							
			2050 RCP 4.5		2050 RCP 8.5		2100 RCP 4.5		2100 RCP 8.5	
Island	Beach name	Maximum Width(m)	Ensembles retreat (m)	Retreat percentage (%)	Ensembles retreat (m)	Retreat percentage (%)	Ensembles retreat (m)	Retreat percentage (%)	Ensembles retreat (m)	Retreat percentage (%)
**St Thomas**	Unknown Beach west of Maggens Bay	6	4.10	68	4.92	82	8.36	100	12.89	100
**St Thomas**	Maggens Bay	33	4.04	12	4.84	15	8.23	25	12.68	38
**St Thomas**	Tutu	7	6.62	95	7.97	100	13.56	100	20.94	100
**St Thomas**	Water Bay	18	13.44	75	16.22	90	27.79	100	43.07	100
**St Thomas**	Sugar Bay	6	2.51	42	3.08	51	5.27	88	8.21	100
**St Thomas**	Cowpet Bay	40	1.59	4	1.93	5	3.34	8	5.21	13
**St Thomas**	Scott Beach 2	7	4.34	62	5.23	75	8.95	100	13.85	100
**St Thomas**	Unknown Beach 18	7	4.46	64	5.40	77	9.30	100	14.46	100
**St Thomas**	Unknown Beach 19	15	1.39	9	1.68	11	2.92	19	4.54	30
**St Thomas**	Prince Ruperts Cove	9	1.89	21	2.28	25	3.94	44	6.12	68
**St John**	Frank Bay	10	1.71	17	2.08	21	3.65	36	5.71	57
**St John**	Galge Cove	14	1.69	12	2.07	15	3.63	26	5.70	41
**St John**	Caneel Bay	10	1.90	19	2.31	23	4.05	40	6.33	63
**St John**	Turtle	17	1.12	7	1.37	8	2.38	14	3.72	22
**St John**	Monte Bay 2	18	1.44	8	1.73	10	2.96	16	4.58	25
**St John**	Monte Bay	7	5.10	73	6.13	88	10.45	100	16.13	100
**St John**	Hult Bay	10	10.99	100	12.98	100	21.28	100	31.52	100
**St John**	Unknown Beach 32	19	1.86	10	2.23	12	3.81	20	5.91	31
**St John**	Chocolate Hole	32	3.53	11	4.19	13	7.25	23	11.22	35
**St John**	Great Cruz Bay	17	3.77	22	4.55	27	7.80	46	12.08	71
**St Croix**	Pelican Cove	6	14.47	100	17.47	100	29.96	100	46.44	100
**St Croix**	Unknown Beach 38	8	15.97	100	19.26	100	32.96	100	51.06	100
**St Croix**	Danish building	8	19.50	100	23.49	100	40.14	100	62.12	100
**St Croix**	Unknown Beach 40	3	27.28	100	36.21	100	56.20	100	86.99	100
**St Croix**	Christiansen Harbor	5	3.32	66	4.03	81	6.96	100	10.82	100
**St Croix**	Chanay	26	2.05	8	2.50	10	4.37	17	6.85	26
**St Croix**	Solitude Bay	5	2.83	57	3.43	69	5.92	100	9.22	100

**St Croix**	Grapetree	22	25.06	100	30.02	100	50.73	100	78.05	100
**St Croix**	Devi	20	4.88	24	5.86	29	9.98	50	15.42	77
**St Croix**	East end marine park	4	15.51	100	18.65	100	31.73	100	49.00	100

Beach_Inventory.kml

A .kml geospatial file is provided along with the submission containing the beaches inventory in the USVI with all the geophysical, socioeconomic characteristics and their respective erodibility, socio-economic and coastal vulnerability index. The user can open the file in Google Earth and navigate the beaches and their characteristics. The inventory contains these characteristics for a total of 191 beaches (out of 210 identified, some of the characteristics were not available for a few of the beaches identified). More specifically the inventory contains information regarding (columns from left to right):

1) Beach ID, The ID is of the beaches counted clockwise, 1-61 for St. Thomas, 62 - 135 for St. John and 136 – 210 for St. Croix, 2) Beach Name, 3) Island, 4) slope, beach linear profile in %, 5) WP, wave power in kW/m, 6) IN, Iribarren number, 7) ESL, extreme sea levels in m, 8) R_hab, natural habitat index, 9) Bus + Hot, number of touristic businesses and hotel in a close proximity of each beach (1km), 10) Visitation, average number of photographs upload in the flickr database in a close proximity of each beach (1km) for the years 2005-2017, 11) width, beach maximum width in m, 12) Res/tial, Residential development of each beach. Number of residential buildings in a close proximity of each beach(1 km), 13) Assets, number of buildings in a very close proximity of each beach (100m), 14) EI, Erodibility Index, 15) SEI, socio-economic importance index, 16) CVI, coastal vulnerability index
